# Cold stress tolerance of the intertidal red alga *Neoporphyra haitanensis*

**DOI:** 10.1186/s12870-022-03507-x

**Published:** 2022-03-14

**Authors:** Shanshan Zhu, Denghui Gu, Caiping Lu, Caixia Zhang, Juanjuan Chen, Rui Yang, Qijun Luo, Tiegan Wang, Peng Zhang, Haimin Chen

**Affiliations:** 1 State Key Laboratory for Managing Biotic and Chemical Threats to the Quality and Safety of Agro-products, Ningbo University, Ningbo, 315211 Zhejiang China; 2 College of Life Sciences, Zhejiang University, Hangzhou, 310058 Zhejiang China; 3 Collaborative Innovation Center for Zhejiang Marine High-efficiency and Healthy Aquaculture, Ningbo University, Ningbo, 315211 Zhejiang China; 4 Zhejiang Mariculture Research Institute, Wenzhou, 325005 China

**Keywords:** *Neoporphyra haitanensis*, Cold stress, Transcriptome

## Abstract

**Background:**

Red algae *Porphyra* sensu *lato* grow naturally in the unfavorable intertidal environment, in which they are exposed to substantial temperature fluctuations. The strategies of *Porphyra* to tolerate cold stress are poorly understood.

**Results:**

Herein, investigations revealed that chilling and freezing induced alterations in the physiological properties, gene transcriptional profiles and metabolite levels in the economically important red algae species, *Neoporphyra haitanensis*. Control samples (kept at 20 °C) were compared to chilled thalli (10 and 4 °C) and to thalli under − 4 °C conditions. Chilling stress did not affect the health or photosynthetic efficiency of gametophytes, but freezing conditions resulted in the arrest of growth, death of some cells and a decrease in photosynthetic activity as calculated by *Fv/Fm*. Transcriptome sequencing analysis revealed that the photosynthetic system was down-regulated along with genes associated with carbon fixation and primary metabolic biosynthesis. Adaptive mechanisms included an increase in unsaturated fatty acids levels to improve membrane fluidity, an increase in floridoside and isofloridoside content to enhance osmotic resistance, and an elevation in levels of some resistance-associated phytohormones (abscisic acid, salicylic acid, and methyl jasmonic acid). These physiochemical alterations occurred together with the upregulation of ribosome biogenesis.

**Conclusions:**

*N. haitanensis* adopts multiple protective mechanisms to maintain homeostasis of cellular physiology in tolerance to cold stress.

**Supplementary Information:**

The online version contains supplementary material available at 10.1186/s12870-022-03507-x.

## Background

The temperature of the sea and air in the intertidal zone fluctuates greatly over a 24-h period. The temperature changes are especially substantial during the seasonal transition between both autumn and winter, and winter and spring. Therefore, algae species that live in the intertidal zone must adapt to survive during temperature change stresses that occur in the intertidal environment [1]. Much is known about high temperature stress responses in macroalgae [2, 3]. However, little is known about how they respond to cold stress. *Porphyra* sensu *lato* are typical intertidal mesophilic red algae, some of which are the most economically important seaweeds in Asia [4]. The primary growth period of *Porphyra* gametophytes in seawater occurs from autumn to the following spring. For example, the cultivation period of *Neoporphyra haitanensis*, which is endemic to China, is from September to February [5]. For *Neopyropia yezoensis*, their cultivation period occurs from November to the following April [6]. During this period, there is a cold winter as well as seasonal changes. In recent years, due to global warming of the climate, the cultivation area of *Porphyra* along the coast of China has been extended northward [7]. However, winter water temperatures in the northern sea can become particularly low and often falls below zero with sea ice forming along the coast. Takahashi et al. found that for *N. yezoensis*, growth almost stops when the temperature is lower than 5 °C [8]. While in a more suitable temperature range (10–20 °C), the growth rate generally accelerates when the temperature increases [9]. As we know from farming experience, the optimal growth temperature for young thalli of *N. haitanensis* is 25–27 °C, while for mature thalli is 20–22 °C. Since cold injury is a limiting factor to plant cultivation [10], it is not known whether this seaweed can grow normally under such low temperatures and what may be their coping mechanisms. To answer this question, we studied the responses of *N. haitanensis* to cold stress in order to aid the development of mass culturing technologies for *Porphyra*.

Cold stress includes chilling (below 15 °C) and freezing (below 0 °C) [11, 12]. In plants, moderate cold stress leads to chlorosis, progressive membrane and oxidative damage. Meanwhile, at temperatures below 3 °C, cell wall arrangement is disrupted by the formation of extracellular ice. Ice formation can lead to physical rupturing of the cell wall, and subsequently cell death. Furthermore, growth and development have been shown to be reduced after ice-formation by a direct inhibition of metabolic reactions and, indirectly, through cold-induced osmotic, oxidative, and energetic stresses [13–15]. To avoid the injuries caused by these effects, *Porphyra* cells are required to adapt with numerous physiological, biochemical, and molecular alterations in order to endure low temperature conditions. These processes have been extensively studied in higher plant species. It has been reported that stress-response related genes [16] and genes associated with the antioxidative system [10] are up-regulated and cryoprotectant osmolyte levels are increased after cold stress, whilst photosynthesis [17], nutrient absorption, and growth have all been shown to be reduced. Lipid compositions have also been observed to be altered to stabilize the membranes [18].

Currently, studies investigating cold stress tolerance in algae mainly focus on single-cell green algae, such as species of Antarctic psychrophilic algae (*Chlamydomonas* sp. *ICE-L* and *Tetrabaena socialis*) [19], *Klebsormidium crenulatum* [20]*,* and *Chlamydomonas reinhardtii* [15]. The freezing of *K. crenulatum* was reported to result in dynamic changes in cellular ultrastructure and cell wall architecture [20]. Valledor et al. observed an accumulation of sugar, alcohols, and other cryoprotectants. Furthermore, levels of unsaturated fatty acids were increased, while oxidative phosphorylation and photosynthesis were partially reduced in response to cold stress [15]. Antarctic psychrophilic algae also exhibited adaptations that included enhanced membrane fluidity, better antioxidant strategies, and improved stability of the photosynthetic apparatus [19].

Currently, several genome-wide studies have revealed various molecular alterations that may contribute to extreme environmental tolerance and survival of algae [21, 22]. Chromosome-level genomes published in recent years have provided important data for the study of cold tolerance of economically important intertidal seaweeds [21, 23]. In the present study, strategies to several levels of cold stress, subdivided into chilling- (10 and 4 °C) and extracellular freezing (− 4 °C) stress, were investigated in *N. haitanensis*. Transcriptomic analysis in correlation with physiological experiments and metabolite measurements were performed and a classical set of responses were identified. These responses included a reduction in photosynthetic activity, fatty acid composition alterations, and some specific responses involving the enhancement of ribosomal biogenesis that are predicted to provide support to the physiological adaptations required for cold temperature survival.

## Results

### Morphological responses of *N. haitanensis* to cold stress

**Fig. 1:**
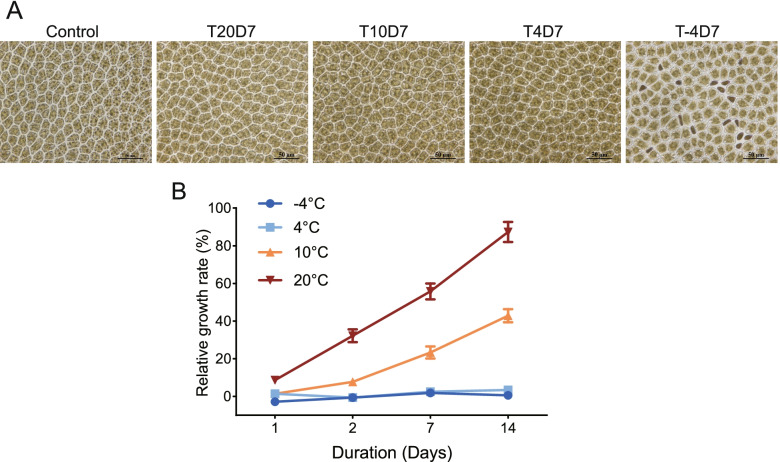
Physiological changes of *Neoporphyra haitanensis* exposed to cold stress. **A** Microscopic changes of cells. Thalli were cultured at − 4, 4, 10 and 20 °C for 7 days (T-4D7, T4D7, T10D7 and T20D7). Samples were examined under a microscope (Scale bar = 50 μm). **B** Effect of cold stress on the growth rate of thalli. *n* = 10

The cellular status of *N. haitanensis* was observed under different low temperatures. It was found that reducing the culturing temperature to 10 or 4 °C had no effect on cell state. Even if the thalli were cultured for 7 days, there was no obvious damage to cells and the overall color was normal. Dropping the cultivation temperature to − 4 °C, during which thalli were immersed in a mixture of ice and water, and culturing for 7 days led to several blenched areas around the edges of the thalli. Microscopic observation of blenched cells showed that the pigment of the cells was weakened, organelles were pyknotic, and some cells had died (Fig. 1A). Thalli cultured at − 4 and 4 °C stopped growing and exhibited no increase in fresh weight within 14 days. However, growth was observed at both 10 °C and 20 °C and was faster at 20 °C (Fig. 1B).

### The overall changes of the transcriptome during cold stress

The Illumina HiSeq 2000 platform was used to analyze the transcription level changes of *N. haitanensis* when cultured under low temperature stress. A total of 2.6 × 10^7^ clean reads with an average length of 150 bp were obtained; 77.55% of the clean reads could be aligned to the *N. haitanensis* genome. There were 11,471 genes in the *N. haitanensis* transcript, of which 8530 genes were expressed (FPKM > 0, Additional file 1). We randomly selected twelve unigenes to validate the RNA-seq analysis by using quantitative real-time PCR (qRT-PCR). The expression of these genes in the transcriptome data were generally consistent with the qRT-PCR results (Additional file 2: Fig. S1).

**Fig. 2:**
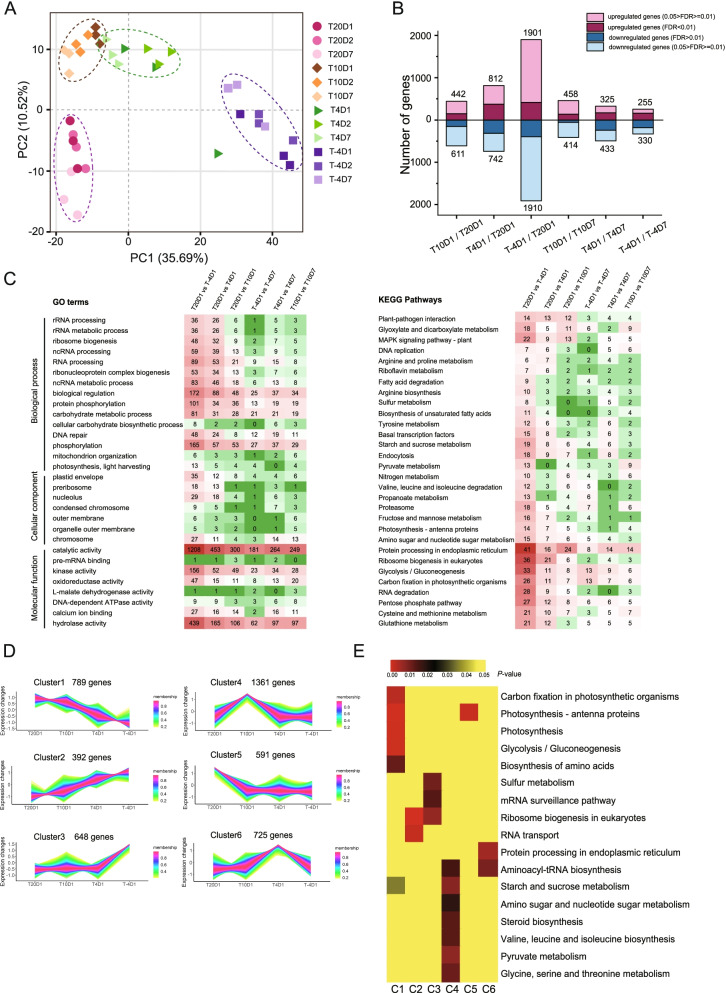
Transcriptional responses of *Neoporphyra haitanensis* to cold stress. **A** Principal components analysis (PCA) of the transcriptomic profile. **B** Number of differentially expressed genes (DEGs) between different temperature groups. **C** Heatmaps of GO and KEGG enrichment of DEGs. **D** Scaled transcript profiles that were identified by the clustering analysis of *N. haitanensis* undergoing cold stress. The DEGs were clustered into six groups using the fuzzy c-means clustering method. The vertical axis shows the normalized FPKM expression values of DEGs. **E** The Heatmap describes significantly enriched KEGG pathways for each cluster of the transcriptome dataset. Thalli were cultured at − 4, 4, 10 and 20 °C (T-4, T4, T10 and T20) for 1, 2 or 7 days (D1, D2, or D7)

Principal component analysis (PCA) analysis found that temperature was the main factor influencing transcription. With a decline in temperature, compared to the group cultured at 20 °C, each subsequent temperature group showed obvious clustering with distances increasing from near to far. The transcription data in the − 4 °C group was the furthest from that in the 20 °C group, indicating the greatest difference (Fig. 2A). Compared with the T20D1, the T − 4D1 group had 3811 differential genes, of which 1901 were up-regulated and 1910 were down-regulated genes (Fig. 2B). Prolonging the incubation time at the set temperatures had little influence on gene transcriptional changes. The data at each time point was generally clustered by temperature groups (Fig. 2A).

The enrichment analysis of identified differentially expressed genes (DEGs) by Gene Ontology (GO) and Kyoto Encyclopedia of Genes and Genomes (KEGG) also showed that the main differences were seen between the different temperature groups. There were also a few different genes between the different cultivation time groups. GO enrichment revealed that significantly enriched biological process terms included: “Biological regulation,” “Phosphorylation,” “Catalytic activity,” “Hydrolase activity,” and “Kinase activity,” indicating that a large number of genes related to regulation changed when encountering cold stress. A total of 112 KEGG pathways were enriched in DEGs, including “Protein processing in endoplasmic reticulum” and “Ribosome biogenesis in eukaryotes” which were all associated with protein synthesis processes; “Glycolysis/Gluconeogenesis,” “Carbon fixation in photosynthetic organisms,” “Photosynthesis-antenna proteins,” “Starch and sucrose metabolism,” and “Pentose phosphate pathway” which were the main pathways relating to photosynthesis and carbohydrate synthesis; “Fatty acid degradation” and “Biosynthesis of unsaturated fatty acids” which were associated with fatty acid metabolism; and “MAPK signaling pathway” and “Basical transcription factors” which were associated with signaling and regulation pathways (Fig. 2C). This indicated that adapting to unfavorable environments is a complex process.

Based on the findings above, that temperature is the main factor influencing the transcriptional differences, we then focused on the analysis of the differential transcriptions of *N. haitanensis* cultured under differing low temperatures for 1 day. Gene-co-expression analysis and the clustering of the 4506 DEGs into six major clusters were conducted (Fig. 2D). The gene expression level in Cluster 1 was down-regulated as the temperature dropped. KEGG enrichment revealed that pathways involved in photosynthesis, carbon fixation, and glycolysis pathways were affected (Fig. 2E). The trend in cluster 2 gene expression was to increase with a decrease in temperature. Most of these DEGs were involved in “Ribosome biogenesis in eukaryotes” and “RNA transport” pathways. The expression levels of DEGs in cluster 3 were significantly up-regulated when the temperature dropped to − 4 °C. Most of these DEGs were related to “Sulfur metabolism,” “mRNA surveillance,” and “Ribosome biogenesis in eukaryotes” pathways. The gene expression levels of cluster 4 were not down- or up-regulated at 10 °C, but when the temperature dropped below 10 °C, genes in this cluster were down-regulated. These genes were mainly involved in the synthesis of various substances, including starch, amino acids, and pyruvate. The DEGs in cluster 5 were significantly down-regulated at 10 °C and remained down-regulated when the temperature decreased further. These DEGs focused on the “Photosynthesis-antenna proteins” pathway. The DEGs in cluster 6 were up-regulated between 20 and 4 °C, but returned to normal levels from 4 °C to − 4 °C. These genes were mainly enriched in the “Aminoacyl-tRNA biogenesis” and “Protein processing in endoplasmic reticulum” pathways (Fig. 2D, E).

### Cold stress suppressed photosynthesis

**Fig. 3:**
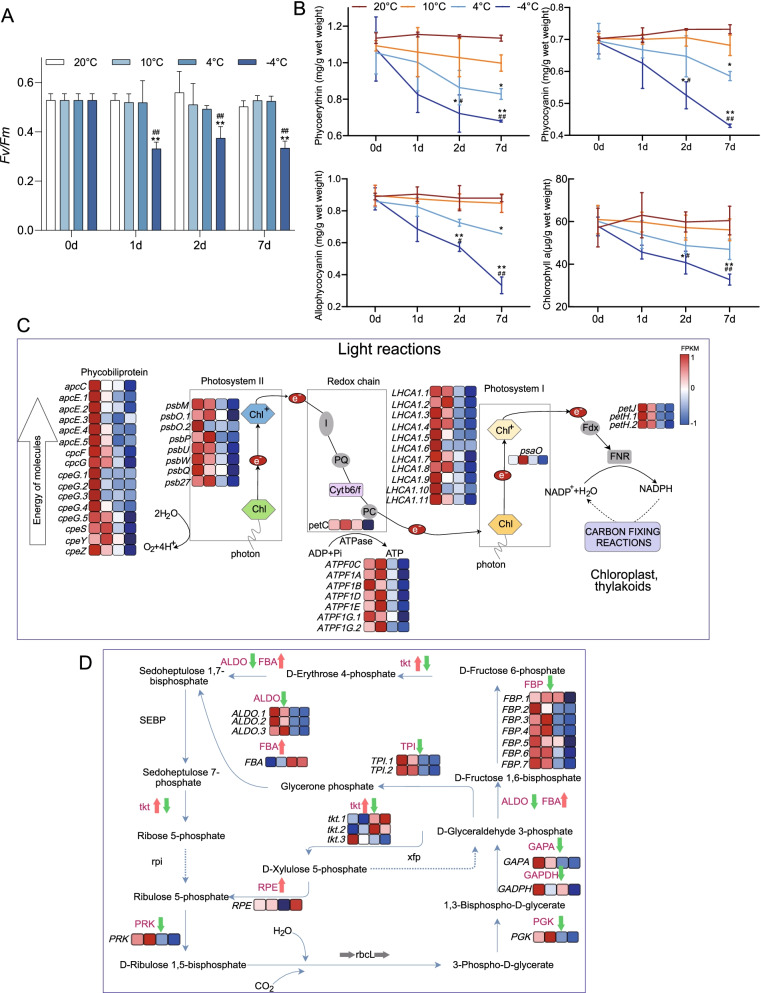
Strategies of *Neoporphyra haitanensis* photosynthetic system responding to cold stress. **A** Photochemical efficiency of *N. haitanensis* during low temperature treatment. Thalli were cultured at − 4, 4, 10 and 20 °C (T-4, T4, T10 and T20) for 1, 2 or 7 days (D1, D2, or D7). ^**^P < 0.01, compared to 20 °C group; ^##^*P* < 0.01, compared to 0 day group. **B** Changes of photosynthetic pigments contents under cold stress. ^*^P < 0.05, ^**^P < 0.01, compared to 20 °C group; ^#^*P* < 0.05, ^##^*P* < 0.01, compared to day-0 group. **C** Transcriptome expression profiles of the *N. haitanensis* photosynthetic system in cold conditions are shown near the pathways as heatmaps. **D** Overview of the Calvin cycle of *N. haitanensis* in response to cold stress. The data shows the transcriptomic profiles from the T20D1, T10D1, T4D1 and T-4D1 groups (from left to right). FPKM values of genes in different groups were used to construct the heatmaps after a z-score normalization (*n* = 3)

The maximum photosynthetic efficiency of *N. haitanensis* did not change when cultured under 10 or 4 °C, even after being cultured for 7 days. However, when compared to the thalli cultured under 20 °C, the freezing temperature of − 4 °C had a great influence on the *Fv/Fm* of thalli, which decreased significantly on the first day by 37.30% (*P* < 0.01). In the following 2 to 7 days, the level of *Fv/Fm* was low, but there was no significant difference compared with the first day of low-temperature culture. This indicated that the photosynthetic efficiency of *N. haitanensis* would rapidly reduce to a certain extent under low temperature, but would not continuously decrease and would still retain a certain photosynthetic capacity at − 4 °C (Fig. 3A).

In addition, we found that the contents of chlorophyll *a* and three phycobiliproteins, including phycoerythrin, phycocyanin and allophycocyanin, did not change significantly at 20 and 10 °C (Fig. 3B). Although they decreased at 4 °C, phycobiliproteins only decreased significantly at 7d (*P* < 0.05). However, these pigments declined significantly at − 4 °C, especially at 2 d and 7 d (*P* < 0.01).

From the above cluster analysis, it can be seen that the photosynthetic system is down-regulated under cold stress. We found 31 genes encoding photosynthesis that were differentially expressed, including phycobiliprotein, photosystem I and II core complex proteins, and ATPase genes. Phycobiliprotein genes, including *apc*, *cpe*, *cpc*, decreased when the temperature dropped to 10 °C. While other genes showed no significant changes at 10 °C, all genes in the photosynthetic system were significantly down-regulated at 4 °C and − 4 °C under cold stress (*P* < 0.05). The lower the temperature, the more obvious the decline (Fig. 3C, Additional file 3).

The down-regulation of the entire photosynthetic system caused by excessive low temperatures reduced the production of NADPH and ATP, in turn affecting the carbon-fixed Calvin cycle. A total of 18 Calvin cycle genes were found to be differentially expressed. Excluding transketolase (*tkt*) genes, all DEGs of the Calvin cycle were down-regulated at 4 °C and particularly at − 4 °C, indicating that the influx and regeneration of carbon during cold stress were also suppressed (Fig. 3D).

### Cold stress contributed to the accumulation of floridoside and isofloridoside in *N. haitanensis*

**Fig. 4:**
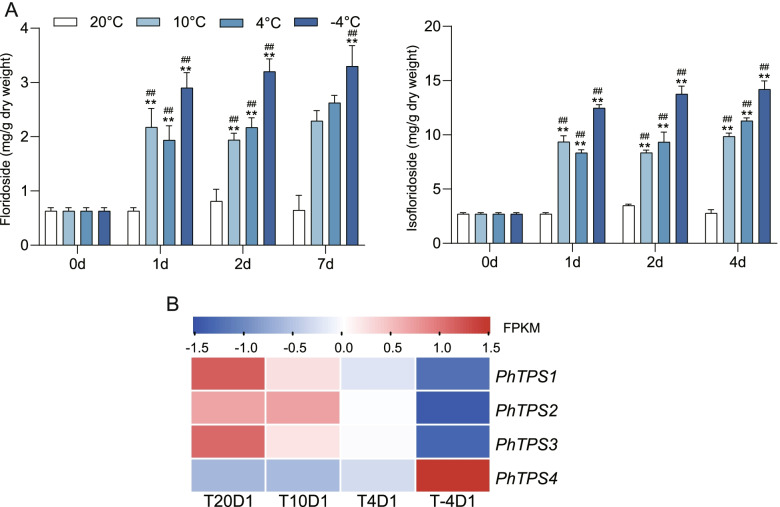
The responses of floridoside and isofloridoside in *Neoporphyra haitanensis* to cold stress. **A** Floridoside and isofloridoside level changes. ^**^P < 0.01, compared to 20 °C group; ^##^*P* < 0.01, compared to Day 0 group. **B** The expression of putative floridoside-6-phosphate synthases genes. Thalli were cultured at − 4, 4, 10 and 20 °C for 1 day (T-4D1, T4D1, T10D1 and T20D1). FPKM values of the genes in different groups were used to construct the heatmap after a z-score normalization (*n* = 3)

In this study, the floridoside and isofloridoside contents of *N. haitanensis* gametophytes under different low temperatures were analyzed (Fig. 4A). Floridoside and isofloridoside are isomers. In *N. haitanensis*, isofloridoside makes up the majority of these isomers with an abundance 4–5 times greater than that of floridoside. Both compounds responded quickly and strongly to cold stress, following a similar change. Their levels rose significantly in the early stages of cooling and remained at higher levels during the subsequent low temperature culture. This increase was more significant with a larger decrease in temperature. When cultured for 1 d, the floridoside contents of thalli at 10 and 4 °C increased by 3.08 and 3.46 times respectively when compared to the contents when cultured at 20 °C (*P* < 0.01). The floridoside content at − 4 °C was 4.6 times that of 20 °C (*P* < 0.01). The isofloridoside content when cultured at − 4 °C for 4 days was 5.11 times higher than when cultured at 20 °C, reaching 14.19 ± 0.78 mg/g (*P* < 0.01).

Four genes annotated as trehalose-6-phosphate synthase that may perform floridoside-6-phosphate synthase function (*PhTPS1–4*) in *N. haitanensis* were found [24]. Of them, only *PhTPS4* was up-regulated when cultured at low temperature, especially at − 4 °C; some other genes were even down-regulated (Fig. 4B).

### The composition of fatty acids in *N. haitanensis* changed with cold stress

A total of 30 fatty acids were detected in *N. haitanensis*, mainly composed of the fatty acids 16:0, 18:0, C22:0, C20:4n6, 20:3n3, C20:5n3. These 6 fatty acids accounted for 80% of the total fatty acid content. Under different low temperature stresses, the fatty acid species did not change, but the proportion of unsaturated fatty acid (UFA) increased significantly (Table 1). Several UFAs increased significantly when the temperature decreased to 10 °C, including several eicosanoic acids 20:3 and 20:4 (*P* < 0.01); there was a further significant increase as the temperatures dropped further. For example, the content of C20:3n3 increased by 27.44% (*P* < 0.01), 22.79% (*P* < 0.05), and 16.19% (*P* < 0.05) respectively at 10 °C, 4 °C, and − 4 °C compared to the levels at 20 °C.

**Table 1:** Fatty acids in Neoporphyra haitanensis under different cold stress (%)

Fatty acids	20 °C	10 °C	4 °C	-4 °C
**C14:0**	0.39 ± 0.02	0.35 ± 0.02	0.37 ± 0.01	0.38 ± 0.03
**C16:0**	16.96 ± 0.01	14.02 ± 1.1	14.00 ± 0.19^**^	14.77 ± 0.08^**^
**C16:1**	0.82 ± 0.1	0.96 ± 0.14	0.96 ± 0.01	0.99 ± 0.15
**C18:0**	12.99 ± 0.53	9.02 ± 0.28^*^	9.47 ± 0.49^*^	10.28 ± 1.03
**C18:1**	2.95 ± 0.27	3.66 ± 0.36	3.58 ± 0.26	3.63 ± 0.48
**C18:2**	3.62 ± 0.07	4.81 ± 0.44	4.48 ± 0.09^*^	4.25 ± 0.22
**C18:3n6**	0.26 ± 0.01	0.34 ± 0.03	0.32 ± 0.01^*^	0.3 ± 0.04
**C20:0**	0.21 ± 0.01	0.14 ± 0.01^*^	0.14 ± 0.02	0.16 ± 0.03
**C20:1**	1.99 ± 0.13	2.58 ± 0.37	2.51 ± 0.07	2.28 ± 0.18
**C20:2**	1.17 ± 0.06	1.49 ± 0.16	1.51 ± 0.06^*^	1.42 ± 0.1
**C20:3n6**	1.83 ± 0.02	2.48 ± 0.09^**^	2.27 ± 0.02^**^	2.23 ± 0.02^**^
**C20:4n6**	6.35 ± 0.15	8.04 ± 0.02^**^	7.75 ± 0.12^*^	7.34 ± 0.06^*^
**C20:3n3**	6.67 ± 0.17	8.5 ± 0.02^**^	8.19 ± 0.13^*^	7.75 ± 0.06^*^
**C20:5n3**	21.82 ± 0.26	21.75 ± 0.31	22.11 ± 0.32	22.03 ± 0.21
**C22:0**	19.23 ± 0.23	19.17 ± 0.27	19.48 ± 0.28	19.42 ± 0.18
**C22:1n9**	1.32 ± 0.09	1.55 ± 0.18	1.59 ± 0.04	1.46 ± 0.19
**UFA**	49.59 ± 0.71	56.73 ± 1.57^*^	55.9 ± 0.06^*^	54.31 ± 1.14^*^
**SFA**	50.41 ± 0.63	43.27 ± 0.87^*^	44.1 ± 0.22^*^	45.69 ± 1.06^*^

Note: ^*^P < 0.05, ^**^P < 0.01, compared with 20 °C

**Fig. 5:**
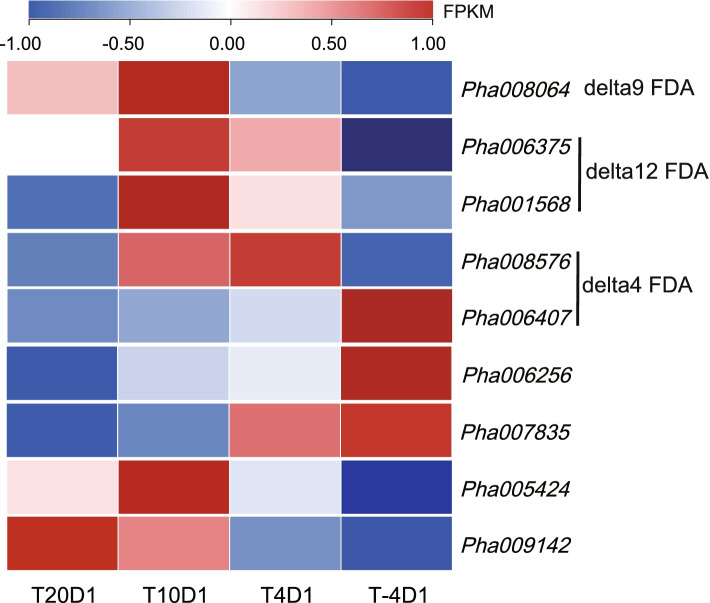
The heatmap of fatty acid desaturase genes of *Neoporphyra haitanensis* when subjected to cold stress. Thalli were cultured at − 4, 4, 10 and 20 °C for 1 day (T-4D1, T4D1, T10D1 and T20D1). FPKM values of genes in different groups were used to construct the heatmap after a z-score normalization (*n* = 3)

A total of 9 genes encoding fatty acid desaturase (FAD) were found in *N. haitanensis* (Fig. 5, Additional file 3), including delta12 (Pha006375, Pha001568), delta9 (Pha008064), and delta4 FAD (Pha008576, Pha006407). Many of these genes were up-regulated when cultured at low temperatures, and most genes were up-regulated when the temperature was lowered to 10 °C. For example, two delta12 genes and delta9 genes were significantly up-regulated (*P* < 0.01). There are 3 FAD genes that continued to be up-regulated as the temperature dropped (Pha006256, Pha007835, and Pha006407); however, many genes that were up-regulated at 10 °C decreased when the temperature dropped to 4 °C. The corresponding saturated fatty acid (SFA) substrates of these enzymes all decreased with the decrease in temperature. When the temperature decreased to 4 °C, the relative contents of 16:0 and 18:0 dropped to 85.55% (*P* < 0.01) and 72.9% (*P* < 0.05) in the 20 °C group, whilst the corresponding product UFAs increased. The unsaturation index UFA/SFA at 10 and 4 °C were significantly higher than that at 20 °C, specifically 1.32 times and 1.28 times higher, respectively (*P* < 0.01).

### Ribosome biogenesis increased during cold stress

**Fig. 6:**
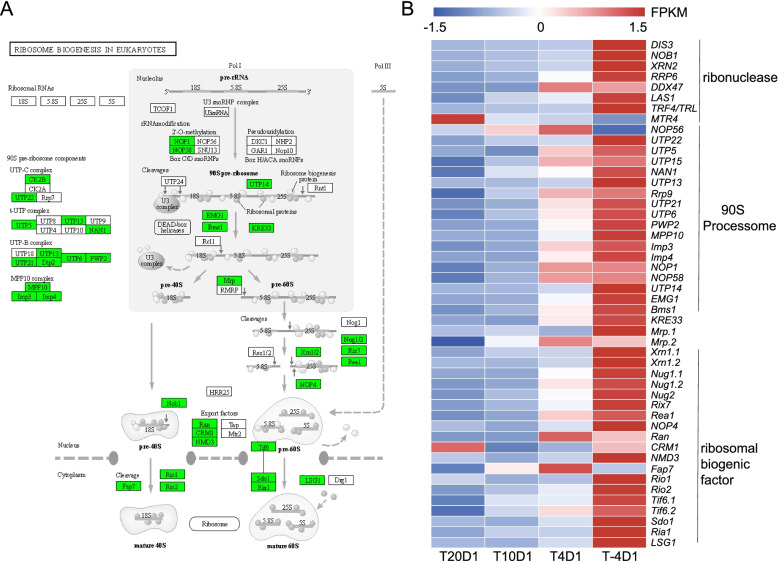
The expression of ribosome biogenesis genes in *Neoporphyra haitanensis* during cold stress. **A** Schematic of ribosomal biogenesis [25–27]. The detected genes are shown in green. **B** Heatmap of the differential expressed ribosome biogenesis genes

Figure 6A is a diagram of the ribosome assembly pathway in eukaryotes [25–27]. There were a large number of differential genes under cold stress enriched in this pathway (Additional file 3). The transcription and processing of rRNA are the rate-limiting steps of ribosomal biogenesis. Eight ribonuclease genes were found in the *N. haitanensis* transcriptome (Fig. 6B). Except for the MTR4 gene (a putative RNA helicase and exosome co-factor), the other genes were all increased as the temperature decreased, especially at − 4 °C. For example, NOB1 (NIN1/RPN12 binding protein 1 homolog) is required for the cleavage of 20Spre-rRNA. Compared to the control level at 20 °C, the expression level at − 4 °C was 5.06 times greater (*P* < 0.01).

In the process of ribosome biogenesis, rRNA precursors, ribosome assembly factors, and small nucleolar ribonucleoprotein particles (snoRNPs) are combined into 90S pre-ribosomes in the nucleolus [28]. Some related homologous genes were found in *N. haitanensis*, including the U3snoRNP and Nop56 genes, Nop58 and Nop1 genes, and the U3 specific proteins Rrp9, Mpp10p, Imp3 and Imp4 genes (Fig. 6). Most genes were up-regulated at low temperatures, and the up-regulation became more pronounced as the temperature decreased.

During the transcription process, the pre-RNA interacts with the ribosomal biogenic factor (RBF), which is necessary for RNA processing and the assembly of ribosomal protein small and large subunits into pre-ribosomes [29]. A total of 72 RBFs were found in *N. haitanensis*, 39 of which were differentially expressed when the temperature was lowered. The expression levels of most of these genes were significantly up-regulated at − 4 °C (*P* < 0.01, Fig. 6B).

### Phytohormones changed when thalli were subjected to cold stress

**Fig. 7:**
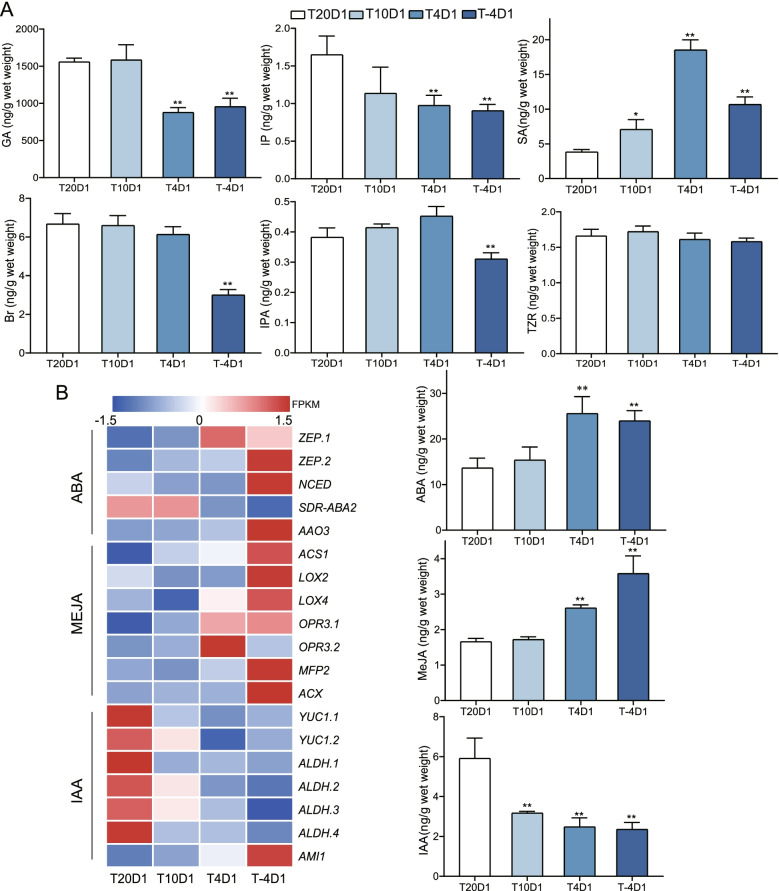
Phytohormone changes in *Neoporphyra haitanensis* subjected to low temperature. **A** The phytohormone contents response to low temperatures. Thalli cultured at − 4, 4, 10 and 20 °C for 1 day (T-4D1, T4D1, T10D1 and T20D1) were collected to determine the levels of gibberellin (GA); N6-(2-isopentenyl) adenine (IP); salicylic acid (SA); brassinolide (Br); N6-(2-isopentyl) adenosine (IPA); trans-zeatin riboside (TZR); abscisic acid (ABA); indolepopionic acid (IAA) and methyl jasmonate (MeJA). **P < 0.01, compared to 20 °C group. **B** Heatmap of phytohormones biosynthesis genes (normalized FPKM) (*n* = 3)

There were 9 phytohormones detected in *N. haitanensis* (Fig. 7A) including: abscisic acid (ABA), indolepopionic acid (IAA), methyl jasmonate (MeJA), salicylic acid (SA), gibberellin (GA), N6-(2-isopentyl)adenosine (IPA), N6-(2-isopentenyl)adenine (IP), trans-zeatin riboside (TZR), and brassinolide (Br). Among them, ABA, MeJA, and SA underwent similar changes. Under cold stress, the levels of these three hormones increased, especially when the temperature dropped to 4 and − 4 °C (*P* < 0.01). Although the SA level dropped when the culture temperature was decreased to − 4 °C, it was still significantly higher than the levels found in the 20 °C group (*P* < 0.01). However, the changes in GA, IP, and IAA were different. The levels of these phytohormones in *N. haitanensis* cultured under 4 and − 4 °C were significantly lower than that of the control group (*P* < 0.01). IAA was especially affected by temperature, its levels at 10 °C, 4 °C, and − 4 °C decreased by 46.36, 58.04, and 60.24% respectively compared to its level at 20 °C (*P* < 0.01). The level of Br also decreased significantly at − 4 °C (*P* < 0.01).

Although not all the genes on the phytohormone biosynthesis pathways were found in *N. haitanensis*, 19 genes which may be involved in the synthesis of ABA, MeJA, and IAA were identified (Additional file 3). The expression level changes of these genes and the corresponding changes to the phytohormone content were similar under cold stress (Fig. 7B).

## Discussion

Temperature stress investigations with respect to *Porphyra* have mainly been concerned with the effects of high temperatures, because the seaweed will encounter short-term seawater warming stress during cultivation. Moreover, there is a requirement for the selection of high-temperature resistant strains of *Porphyra* for cultivation and farming [3, 5]. But *Porphyra* are cold-tolerant seaweeds and their growth period spans the winter. Several papers have mentioned that the thalli can grow well in sea water below 10 °C [5, 30, 31]. Sometimes, the sea water in which seaweeds grow will freeze in winter, and the surface of the seaweeds can also freeze. In addition, cold storage technology has been developed for the cultivation of *N. haitanensis* and *N. yezoensis*. Thalli on nets can be stored for a long time at − 20 °C when properly dehydrated. Even after 1 year of cold storage, the young thalli on the net can continue to grow when they are returned to the sea [30]. However, to our knowledge, few studied cold stress tolerance in seaweed.

The growth characteristics of *N. haitanensis* under chilling and freezing temperatures were different. When *N. haitanensis* was cultured under 10 °C or 4 °C for 7 days, the changes of the thalli and cells were not obvious, without visible damage. *Fv/Fm* also remained at normal levels at 10 °C and 4 °C, indicating that the alga can tolerate temperatures as low as 4 °C and the maximum potential quantum efficiency of PSII remained stable. But at − 4 °C, apparent damage to the thalli including bleached thallus tips and some dead cells appeared, indicating that the freezing stressed *N. haitanensis*. No cell rupture under the freezing conditions was observed, suggesting that *N. haitanensis* had a tolerance strategy to limit ice formation and protect biological macromolecules from damage, similar to plants [32]. Transcriptome analyses revealed that gene transcriptions varied as soon as the temperature dropped. When the temperature dropped to 10 °C, there was an obvious clustering distance from the 20 °C group. But substantial changes were observed when − 4 °C freezing stress was applied, with the highest number of DEGs. Upon analysis of transcriptome profiles, we noted that low temperatures mainly affected the photosynthetic system and the carbon assimilation and sugar metabolism pathways. Photosynthesis is a temperature-sensitive energy transformation process that is necessary for the conversion of light energy into chemical energy. Photosynthetic electron transport is driven by the absorption of light energy, which is converted by transfer through the PSII, Cytochrome b6f, and photosystem I (PSI) [33]. An energy imbalance occurs as a consequence of exposure to low temperature and constant light, which subsequently induces the generation of reactive oxygen species resulting in cell damage [34, 35]. Several mechanisms for mitigating stress-induced energy imbalances have been identified with most involving a reduction in light-harvesting capacity [34]. We deduced that at lower temperatures, in order to combat the energy imbalance, a down-regulation of light-harvesting was adopted via reductions in the production of phycobiliprotein at the transcription level. Thereby photooxidative damage was minimized. We observed a decrease in phycobiliprotein contents at 4 °C, especially at − 4 °C. Moreover, when the temperature was as low as 4 °C, or even − 4 °C, not only was phycobiliprotein production fully inhibited, the entire electron transport chain system and even the production of ATP were down-regulated, thereby maximizing control of oxidative damage caused by imbalances in the electron transport system. When plants or green algae are subjected to low temperature stress, the antioxidant system is generally enhanced to protect cells from the oxidative toxic effects [18, 19]. However, in *N. haitanensis*, we did not observe an obvious overall up-regulation of multiple antioxidant enzymes, indicating that in the case of lower temperatures, an inhibition of the entire photosynthetic system was undertaken by *N. haitanensis* and was used as the main strategy to mitigate photooxidative damage and protect cells. Interestingly, no color change of thalli after 7-day treatment under 4 °C was observed, but the expression levels of genes encoding phycobiliproteins were decreased. In addition, the transcript levels of genes encoding PSII psb subunits were down-regulated at 4 °C, but *Fv/Fm* was not affected. The underlying causes of these observations need to be further studied.

A down-regulation of the photosynthetic system subsequently affected the Calvin cycle and inhibited carbon fixation. Pathways such as carbohydrate, amino acid, and pyruvate metabolism were also down-regulated, indicating that basal metabolic activities were affected by low temperatures and were inhibited to control cell energy loss. The levels of low molecular weight carbohydrates floridoside and isofloridoside, direct products of photosynthesis assimilation [36], were significantly increased when *N. haitanensis* was subjected to cold stress. This response was rapid and floridoside and isofloridoside levels increased dramatically, especially at − 4 °C. In the four putative floridoside-6-phosphate synthases genes (*PhTPS1–4*), only *PhTPS4* was up-regulated under cold stress, suggesting that these genes may have a division of work. PhTPS4 is involved in the biosynthesis of floridoside and isofloridoside, with isofloridoside being the main product [24]. It is also possible that in order to respond quickly to low temperatures, cells use existing enzymes and substrates to rapidly synthesize isofloridoside. Since floridoside and isofloridoside are also involved in the short-term preparation of carbon pools in red algae cells, they can be synthesized during photosynthesis or synthesized from endogenous reserves of other polysaccharides, such as floridean starch [37]. Therefore, it is estimated that during cold stress, algae cells undergo internal processing, leading to a redistribution of carbon. Although *Porphyra* lacks sucrose or trehalose, which are related to low temperature adaptation in plants [38], floridoside and isofloridoside act as substitutes and provide a similar function. Floridoside and isofloridoside are important osmotic pressure regulators in red algae, and also provide an antioxidant effect [39]. However, there may be differences in the function of floridoside and isofloridoside. Most red algae use floridoside as an osmotic pressure regulator. For example, *Bangia atropurpurea*, *Gracilaria sordida*, and *Grateloupia doryphore* accumulate floridoside in a hyperosmostic environment [39–41]. However, there are exceptions; *Poterioochromonas malhamensis* and *Porphyra columbina* use isofloridoside as their main osmotic pressure regulator [39, 42]. In this study, when *N. haitanensis* was subjected to cold stress, isofloridoside accumulated at high levels, acting as the main regulator. Since the level of isofloridoside in *N. haitanensis* is much higher than that of floridoside, it can be concluded that the incremental changes to a small amount of floridoside are not used to control osmotic pressure adjustment. The further accumulation of isofloridoside can effectively stabilize the macromolecular substances and membrane structures in cells at low temperatures, preventing them from being damaged by cold stress.

The transcriptome profile showed a significant enrichment of “Fatty acid degradation” and “Biosynthesis of unsaturated fatty acids”. The fatty acids were also observed to undergo significant changes at low temperatures, with a significant increase in 20:3 and 20:4 to the detriment of C16:0 and C18:0. Many FDA genes were up-regulated when the temperature was lowered, whilst some were down-regulated when the temperature was lowered to 10 °C, these include delta12 and delta9 fatty acid desaturase. delta12 can combine with ω3 desaturase to catalyze saturated fatty acid C18:0 and generate C18:2 and C18:3 [43]. delta9 fatty acid desaturase can catalyze C16:0 and C18:0 as substrates to form a double bond, resulting in C16:1 and C18:1 production [43]. These results indicate that *N. haitanensis* might reprogram FA composition by adjusting FA desaturation systems to adapt to cold temperatures. The desaturation of fatty acid is crucial for preserving cell energy states and membrane fluidity [15], which is related to cell function. Increases in fatty acid unsaturation could increase membrane fluidity and decrease the temperature at which the transition from a liquid-crystalline to a gel phase takes place [19]. There is a positive relationship between high levels of polyunsaturated fatty acids and the ability to adapt to low temperatures [44]. The results of this experiment revealed that the increase of fatty acid desaturation may be one strategy associated with the tolerance of *N. haitanensis* to cold environments.

In the transcriptomic profile, most of the genes related to ribosome biogenesis were up-regulated. Hang et al. believed that chilling stress affects rRNA biogenesis at the step of pre-rRNA processing in *Oryza sativa* L., resulting in a reduction of pre-rRNA production and the assembly of ribosomes to reduce energy consumption [45]. Usually, when exposed to cold stress, it is necessary for an organism to reduce energy-intensive processes to improve the tolerance and survival of cells. However, the opposite findings can be seen in *N. haitanensis*. When subjected to cold stress, *N. haitanensis* increased expression of endoribonuclease and exonuclease genes which are involved in rRNA transcription. The homologous protein genes involved in the assembly of 90S pre-ribosome and a large number of RBF genes required for 35S pre-rRNA splicing and assemble were up-regulated, especially at − 4 °C. Therefore, their up-regulation will undoubtedly accelerate the assembly of ribosomes. We deduced that under cold stress, *N. haitanensis* kept the activity of ribosome assembly even though it will consume high energy, presumably in preparation for protein translation. When temperature dropped from 20 to 4 °C, the “aminoacyl-tRNA biogenesis and protein processing in endoplasmic reticulum” pathway was significantly enriched and up-regulated, which also indicated that protein synthesis was accelerated. This may be related to the tolerance of the algae to the intertidal environment. It is possible that the acceleration of protein synthesis due to the rapid decrease in temperature is necessary for repairing damage caused by cold stress, as well as for the synthesis of specific low molecular weight proteins that increase the cold resistance of plants [46, 47].

Phytohormones play important regulatory roles in plant growth, development, and stress resistance. A total of 9 hormones were detected in *N. haitanensis*, among which GA, Br, IP, and IAA are growth phytohormones which can promote cell division and growth [48]. Achard et al. found that ABA can promote the accumulation of the DELLA protein and improve the cold tolerance of plants [49]. In this study, the levels of GA, Br, IP, and IAA in *N. haitanensis* decreased significantly upon exposure to cold stress indicating that seaweed can adapt to the cold environment by regulating phytohormones and slowing down growth. However, several phytohormones related to stress resistance, such as ABA, MeJA, and SA, were significantly increased at low temperatures. The role of ABA in plant cold stress signals has been thoroughly investigated and ABA overexpression can improve cold tolerance. SA can improve the osmotic regulation and antioxidant capacity of plants. Dong et al. reported that applying exogenous SA improved the cold tolerance of cucumbers [50], and Yang et al. found that SA accumulation in peaches reduced cold damage [51]. It can be seen that *N. haitanensis* behaves similarly to plants regarding the regulation of cold-resistant phytohormones.

## Conclusions

**Fig. 8:**
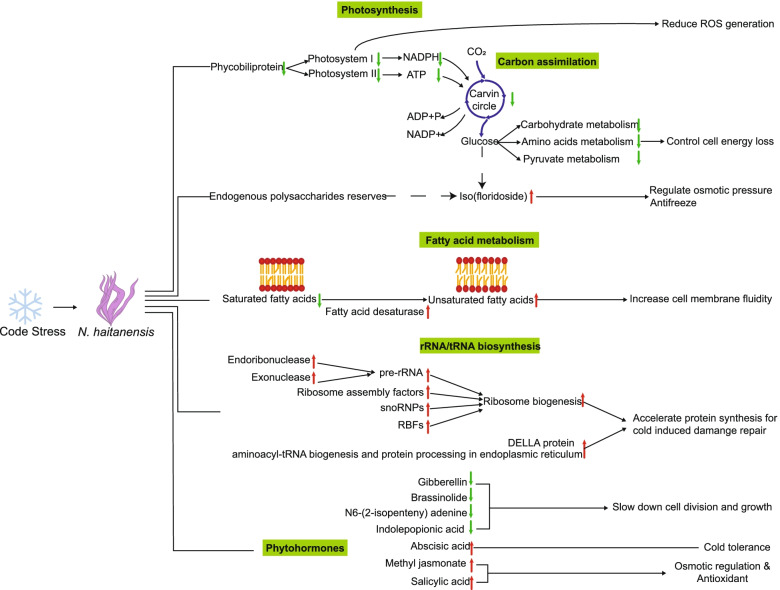
The responses of *Neoporphyra haitanensis* to cold stress

As a high-tide seaweed, *N. haitanensis* is highly tolerant of non-freezing low temperatures, which does not cause cell death or a decrease in photosynthetic efficiency. However, changes at the transcriptional level are apparent when the temperature drops. Down-regulation of the photosynthetic system under cold stress drives the inhibition of carbon fixation and basic substance synthesis. At the same time, the reduction of a variety of growth phytohormones also regulates the growth of seaweed at low temperatures, thereby saving energy. The increase in the degree of fatty acid unsaturation and the increase in isofloridoside content enhances fluidity and the osmotic pressure resistance of the membrane, which helps the seaweed resist cell damage caused by low temperatures. The enhancement of ribosomal biogenesis leads to the acceleration of protein synthesis in response to rapid temperature changes (Fig. 8). In conclusion, this study provides insights into the strategies of *N. haitanensis* to tolerate cold stress.

## Methods

### Sample preparation and treatment

*N. haitanensis* cultivar ZD-1 (developed by our group and registered by the Ministry of Agriculture and Rural Affairs, P. R. China) were used in this study. We collected mature materials from the Experimental Station at the coast of Xiangshan harbor (E 121.85, N 29.66) in Zhejiang province, China in October 2018, and the formal identification of the plant material was carried out by Prof Qijun Luo. No voucher specimen has been deposited in a genebank and no special permission is needed to study this species. Sampling was permitted by the local government (Xiangshan County Government) and the local department of fisheries (Ningbo Ocean & Fishery Bureau).

Thalli were rinsed with sterilized seawater (natural seawater boiled and filtered through 0.22 μm ultrafiltration membrane) and treated with 0.7%KI for 10 min [52]. Thalli with normal coloration and no decay were selected and divided into 4 groups (*n* = 10). Samples from four groups were immersed in sterilized seawater and cultured in constant incubators with temperatures kept at − 4, 4, 10, and 20 °C throughout the whole period (groups of T-4, T4, T10 and T20). The seawater temperature of each sample was constantly monitored. Ice bags were put into the culture solution of the T-4 group to help to keep the stable temperature. The sterilized seawater was changed every other day. Cell status was recorded microscopically on day 7.

During the cultivation, the growth rates were determined. On 1 d, 2 d, 7 d, and 14 d (D1, D2, D7 and D14), the fresh weights of thalli were recorded. Relative growth rate (RGR, %/d) was calculated as follows: RGR = (N_t_-N_0_)/N_0_ × 100%. N_0_ is the initial fresh weight, and N_t_ is the fresh weight after t days.

The samples were collected at 1 d, 2 d, and 7 d. Surface moisture was removed with filter paper and samples were snap-frozen in liquid nitrogen and stored at − 80 °C until analysis. A small amount of each sample was lyophilized for metabolite detection. Three independent biological samples representing each treatment group were harvested for transcriptomic analysis.

### Determination of photosynthetic parameters

The chlorophyll fluorescence parameters of *N. haitanensis* thalli after exposure to different cooling and freezing temperatures for 1 day were recorded using a MAXI IMAGING-PAM system (Walz, Germany) (*n* = 3). The maximum quantum efficiency of the photosystem II (PSII) photochemistry (*Fv/Fm*) was measured at the cell surface immediately after the dark adaptation.

### Photosynthetic pigments measurement

0.1 g frozen fresh samples (in powder form) were homogenized with 8 mL of cold 0.1 M PBS (pH 7.0) and extracted for 10 min with ultrasonication, before placed under − 20 °C for 2 h. The extract was centrifuged at 10,000×g for 5 min. The phycobiliproteins were detected and calculated following the method by Bennet and Bogorad [53], and chlorophyll *a* levels were calculated following the method by Wellburn [54].

### Analysis of free polyunsaturated fatty acids

Frozen powdered samples (50 mg) were ultrasonically extracted by incubation in 1 mL chloroform: methanol (2:1) mixture three times. The resultant liquid solutions were then mixed with 2 mL of 1% sulfuric acid in methanol for esterification at 80 °C for 30 min. The samples were further dried under a stream of N_2_ before being redissolved in hexane and analyzed using a QP2010 GC-MS (Shimadzu, Japan). GC analysis was performed using a TG-FAME silica capillary column (50 m × 0.25 mm × 0.20 μm, Thermo Inc.). The temperature of the injector was 250 °C, the helium carrier gas flow rate was 0.63 mL min^− 1^, and the pre-column pressure was 51.6 kPa. After injection, the oven temperature was held at 80 °C for 1 min, raised to 160 °C at 20 °C min^− 1^, held for 1.5 min and then raised to 196 °C at 3 °C min^− 1^ before being held for 8.5 min, with a final increase to 250 °C at 20 °C min^− 1^ that was held for 3 min. The mass spectrometer was operated in electron compact mode with 1 kV of electron energy. The ion source and interface temperature were 200 and 250 °C, respectively. Masses between the range of m/z 50 to 750 were scanned and fatty acids were quantified from standard curves obtained by measuring the peak areas of authentic standards.

### The quantitation of floridoside and isofloridoside in *N. haitanensis*

One hundred milligrams of freeze-dried samples from each group were extracted by incubation in 1 mL methanol: ddH_2_O (3: 1) 3 times and centrifuged. The supernatant was used for floridoside and isofloridoside analysis on a TSQ Quantum Access analysis system (Thermo Fisher Scientific, USA) using a Hypersil Gold C18 analytical column (100 × 2.1 mm, 3 μm, Thermo Fisher Scientific). 10% 10 mM CH_3_COONH_4_ and 90% acetonitrile were used for isocratic elution. The flow rate was 0.3 ml min^− 1^. The HPLC-MS was operated in the positive ionization mode with the data acquisition mode set to selected reaction monitoring (SRM) for quantification. The transitions monitored for floridoside and isofloridoside in SRM mode were m/z 253 to 119 (20 eV) and m/z 253 to 89 (21 eV). Ions at m/z 89 and 119 were utilized as quantitative ions. The calibration curves for their quantification were constructed using standard compounds extracted directly from *N. haitanensis* as was performed in Chen et al. [55].

### Analysis of phytohormones by LC-MS

The method for phytohormone detection followed the reported procedures with modification [56, 57]. Five hundred milligrams of frozen fresh *N. haitanensis* samples were ground to powder using liquid nitrogen and then extracted with 5 mL MeOH: H_2_O: HCOOH (15: 4: 1) containing 0.5% butylated hydroxytoluene by ultrasonication for 10 min. The processed samples were next stored at − 20 °C in the dark for 16 h. After centrifugation, the residue was re-extracted twice. The obtained supernatants were dried and dissolved in 500 μL of MeOH: H_2_O: CH_3_COOH (90: 10: 0.05) for LC-MS analysis on the Finnigan Surveror and TSQ Quantum Access system equipped with ESI-MS. A hypersil gold C18 column (100 mm × 2.1 mm × 1.7 μm) was used at 30 °C at a flow rate of 0.3 mL min^− 1^. Mobile phase A was 10 mmol L^− 1^ ammonium acetate and 0.2% methanol, and mobile phase B was 100% methanol. Elution was performed with a 20-min 15–100% methanol gradient, maintained at 100% for 5 min before returning to 10% methanol over a 1 min period. 10% methanol was then maintained for 10 min.

The ionization conditions were: sheath gas pressure (N_2_): flow-rate of 30 L min^− 1^; aux gas pressure (N_2_): flow-rate of five Abs; spray voltage: 2500 V; vaporizer temperature: 300 °C; capillary temperature: 32 °C. The collision gas was argon and the gas pressure was 1.5 mTorr. The resolution of Q1 and Q3 was set to 0.7 Da. A series of m/z values were monitored in SRM mode using a time program. The MS was operated in negative mode. The commercial standards of phytohormones were purchased from Sigma-Aldrich (St. Louis, MO, USA) to establish the quantitative standard curves. The quantities of hormones in the samples were determined by comparing the retention time and MS information with the standards [58].

### RNA isolation, library construction, and Illumina sequencing

Three replicates of thalli samples from each different treatment group were collected and snap-frozen in liquid nitrogen before subsequent RNA extraction using a Plant RNA extraction Kit (R6827, OMEGA). Each sample at a concentration greater than 600 ng μL^− 1^ was submitted for transcriptome sequencing. RNA-seq libraries were prepared using the Illumina mRNA-seq Library Preparation kit (Illumina) and sequenced on the Illumina HiSeq platform.

### RNA-seq data analysis

After filtering low quality reads, the clean reads were mapped to the *N. haitanensis* genome assembly using HISAT2. The StringTie program [59] was used to predict new transcripts and these were combined with genome annotations to obtain the final transcriptome set. Reads were mapped to the transcriptome dataset using bowtie2 and quantified using RSEM. Correlations between samples and replicates were examined using PCA performed by the SIMCA-P software package (www.umetrics.com). FPKM was used to calculate the transcripts of all the DEGs. DEseq2 (v.1.22.2) was used to identify DEGs that were defined as those with |log2(fold change)| > 1 and FDR significance score (padj) < 0.05. KEGG annotation enrichment was performed on the DEGs using hypergeometric tests. Hyper-geometric distribution was used to identify enrichment of GO annotations. GO items with FDR ≤ 0.05 were selected as those of significant enrichment. Heatmaps were constructed using Euclidian distances and complete linkage clustering with the pheatmap package for R. To compare differences among treatments, the processed transcriptomic datasets were quantile normalized, transformed, and clustered with hierarchical clustering. Differentially expressed transcripts for each cluster were then evaluated for enrichment of pathways in the KEGG (www.kegg.jp) databases. Statistical significance (*P* values) for each pathway were obtained using the hypergeometric test algorithm. The abundances of pathways with *P* < 0.05 were visualized with a heatmap constructed with TBTools (https://github.com/CJ-Chen/TBtools/releases). Univariate analysis of variance (ANOVA) tests was used to determine the significance of differences in relative contents between different groups.

### qRT-PCR validation

One mg total RNA was used for first-strand cDNA synthesis according to the protocol supplied with PrimeScript™ RT Master Mix (TaKaRa Biotechnology Co., Ltd., Dalian, China) and qRT-PCR amplification reactions were performed using a LightCycler®96 Real-Time PCR System (Roche, Basel, Switzerland). Primers used are listed in Additional file 4: Table S1. Relative expression was calculated using the 2^-ΔΔCt^ method with *β-actin* as the reference gene.

### Statistical analysis

For each experiment, three biological replicates were prepared for each group. The results were presented as the mean ± standard deviation (SD). Statistical significance was evaluated based on a Student *t* tests and Pearson correlation analysis. Data acquired were analyzed using SPSS version 16.0. Differences between groups were considered statistically significant if *P* < 0.05.

## Supplementary Information

**Additional file 1.** Normalized transcriptome data, expressed by FPKM.

**Additional file 2: Figure S1.** Validate the expression of twelve randomly selected unigenes by qRT-PCR. Error bars indicate standard errors of the means (*n* = 3).

**Additional file 3.** Important DEGs, expressed by FPKM.

**Additional file 4: Table S1.** The primers for qRT-PCR.

## Data Availability

The transcriptomic dataset analyzed during the current study are available in the SRA databases under the umbrella BioProject Accession PRJNA772027.

## Abbreviations

DEG: Differentially expressed genes
GO: Gene Ontology
KEGG: Kyoto Encclopedia of Genes and Genomes
PCA: Principal component analysis
UFA: Unsaturated fatty acid
FAD: Fatty acid desaturase
SFA: Saturated fatty acid
RBF: Ribosome biogenesis factors
ABA: Abscisic acid
IAA: Indolepopionic acid
MeJA: Methyl jasmonate
SA: Salicylic acid
GA: Gibberellin
IPA: N6-(2-isopentyl) adenosine
IP: N6-(2-isopentenyl) adenine
TZR: Trans-zeatin riboside
Br: Brassinolide
PSI: Photosystem I
PSII: Photosystem II
