# Supplementary material for: Cold stress tolerance of the intertidal red alga Neoporphyra haitanensis
Source: BMC Plant Biol. 2022 Mar 14;22:114. doi: 10.1186/s12870-022-03507-x (PMC8919617; doi:10.1186/s12870-022-03507-x)
Supplement: Supplementary file 4 — Additional file 4: Table S1. The primers for qRT-PCR. [file 12870_2022_3507_MOESM4_ESM.pdf]

Additional Table S1 The primers for qRT-PCR.

Primers	Sequence (5'→3')	PCR products (bp)
PhNOG1-F	CCCTTTTACGCCGACCTCTT	195
PhNOG1-R	TGAGCATCCGCACATAGTCC	
PhDDX27-F	TTTTTCGCCCAAAAGCGGTC	158
PhDDX27-R	TCCGTGGCGAGCAAGAAG	
PhFAD2-F	GCGGAGTTGTTGGGTATTGC	136
PhFAD2-R	CCTTGGCCTCAAAAATGGGC	
PhLHCA1-F	CGACATCAAGTTCCTGCGTG	200
PhLHCA1-R	AGACAAACTCAACCAGGCCG	
PhpetH-F	ACTGATGCTGAGGGCAACAA	154
PhpetH-R	GCTCCCCAAACATCTCGTCA	
PhDHAR2-F	GTCAACACGCTCGTCAAACC	117
PhDHAR2-R	CAGGTAGGTGACGATGTGCG	
PhthrS-F	CCCACATCTTTTGCTTGCCC	103
PhthrS-R	ATCTCATACTCGCGGAAGCC	
PhpheS-F	TTTGACGACCTCGGCGTG	103
PhpheS-R	TGTGGGCGGTCATGTGTG	
PhRPL12-F	CGAATATGCCCAAAAGGCGG	166
PhRPL12-R	CGAAATCACCAGCCCAGAGT	
PhGADPH-F	TCGTCGGTGTTTGCATCGTC	197
PhGADPH-R	TCAATGCCCGCAAGGATGTC	
PhPDHD-F	TCAAAGTCACCTGCGTGGAG	102
PhPDHD-R	CATACAGGTGCGACGAGTGA	
PhIDH3-F	TCTCAACCCGTTTCCCCTTG	175
PhIDH3-R	GGGTAGACCGTCCTCGTAGT	
Phβ-actin-F	GGTGGTGATTGACAATGGGTCT	193
Phβ-actin-R	CAGCGGGTACTTGATGAGCAG	
